# Scarlet Fever Scare

**Published:** 1893-06

**Authors:** 


### SCARLET FEVER SCARE.

THE laity of Atlanta is just now excited about a supposed epidemic of Scarlet Fever, and one of the public schools of the city has been closed on account of cases occurring among its pupils.

We sympathize with the public in its fear of so malignant a disease, but we deprecate the tendency shown by some of our professional brethren to create a panic by citing through the secular press the location of a few scarlet fever patients.

The closure of the school was probably wise, and we think that the city would do well to pass an ordinance requiring a red flag to be exhibited from houses in which scarlet fever or diphtheria existed, thus preventing visitors and friends from unknowingly exposing themselves.

Isolation of the patient, both during the disease and until all desquamation has ceased, is the best preventive : fumigation and the use of antiseptics is of minor importance and creates in the minds of the public a feeling of security more fancied than real.

The prevalence of epidemics of Scarlatina is largely dependent upon personal intercourse, all other conditions are of minor importance. The clothing worn by the patient, the bed linen, etc., should either be destroyed or subjected to some certain disinfecting process.

Epidemics of Scarlatina occur most often in the fall and winter, and the condition of the weather appears to have little or no influence upon an epidemic.

We believe it to be the duty of the physician to instruct, as far as possible the public, regarding necessary precautions to prevent the spread of disease, but we also believe that he should do all in his power to prevent panics. Nothing is so demoralizing to a communinty as to become panic stricken in time of disease.

Atlanta has been most fortunate in that she has been singularly free from epidemics of all kinds. Scarlet Fever has never been epidemic in the city, and we see no reason why it should now become so. Atlanta is a city, a large and growing city, and necessarily will have, from time to time, cases of contagious and infectious diseases within her limits, but with her location and surroundings, and proper and efficient sanitary regulations, we can see no reason why she should not maintain her envied reputation for healthfulness among the cities of the South.

